# Non-Standard Genetic Codes Define New Concepts for Protein Engineering

**DOI:** 10.3390/life5041610

**Published:** 2015-11-10

**Authors:** Ana R. Bezerra, Ana R. Guimarães, Manuel A. S. Santos

**Affiliations:** Health Sciences Department, Institute for Biomedicine—iBiMED, University of Aveiro, Campus de Santiago, Aveiro 3810-193, Portugal; armbezerra@ua.pt (A.R.B.); rita.guimaraes@ua.pt (A.R.G.)

**Keywords:** genetic code, evolution, codon reassignment, amino acids, biotechnology

## Abstract

The essential feature of the genetic code is the strict one-to-one correspondence between codons and amino acids. The canonical code consists of three stop codons and 61 sense codons that encode 20% of the amino acid repertoire observed in nature. It was originally designated as immutable and universal due to its conservation in most organisms, but sequencing of genes from the human mitochondrial genomes revealed deviations in codon assignments. Since then, alternative codes have been reported in both nuclear and mitochondrial genomes and genetic code engineering has become an important research field. Here, we review the most recent concepts arising from the study of natural non-standard genetic codes with special emphasis on codon re-assignment strategies that are relevant to engineering genetic code in the laboratory. Recent tools for synthetic biology and current attempts to engineer new codes for incorporation of non-standard amino acids are also reviewed in this article.

## 1. Introduction

The genetic code maps 64 codons onto a set of 20 amino acids plus the translational stop signal [1]. These codon-to-amino acid assignments are established by 20 aminoacyl-tRNA synthetases (AARSs) that recognize, activate and charge 20 proteinaceous amino acids onto tRNAs. Aminoacyl tRNAs are then transferred to the ribosome where their three letter anticodons read the three letter codons of messenger RNAs (mRNA) [2]. Although the genetic code is almost universal, 34 alterations in nuclear and organellar genomes (Table 1) from bacterial to eukaryotic species have been discovered [3]. The majority of these codon reassignments involve sense to nonsense codon changes (or *vice versa*) and occur in mitochondria. Only one nuclear sense-to-sense alteration is known so far, namely the reassignment of the CUG codon from leucine to serine in several fungal species of the CTG clade [4,5]. Among code variants involving stop codons are glutamine and cysteine codons of certain ciliates [6] and the tryptophan codon of *Mycoplasma* [7]. Some of these reassignments involve codons whose identities change multiple times in closely related phylogenetic lineages suggesting that certain taxonomic groups (e.g., the ciliates) are more prone to codon reassignment than others [8]. Additionally, two non-canonical amino acids are naturally incorporated into the genetic code, namely selenocysteine, which is inserted at specific UGA sites in a wide range of prokaryotes and eukaryotes [9,10] and pyrrolysine in the archeon *Methanosarcina barkeri* at selected UAG sites [11,12].

These alterations provide insight into the evolution of the genetic code and highlight new concepts that can be used to manipulate protein function for basic and applied research purposes. In recent years, non-canonical amino acids have been incorporated into proteins *in vivo* using orthogonal aminoacyl-tRNA synthetase/tRNA pairs and nonsense codons. More than 100 unnatural amino acids have been incorporated into proteins of numerous organisms, such as *Escherichia coli* [13,14,15], *Saccharomyces cerevisiae* [13], mammalian cells [13,14], *Shigella* [15], *Salmonella* [15], *Mycobacterium tuberculosis* [16], *Drosophila melanogaster* [13], *Caenorhabditis elegans* [13,17], *Bombyx mori* [18] and *Arabidopsis thaliana* [14]. High level misincorporation of canonical amino acids has also been reported. UAG stop codons have been reassigned to glutamine (Gln) and tyrosine (Tyr) in a modified *E. coli* strain lacking both UAGs in essential genes and the release factor-1 (RF1) which recognizes UAGs [19]. Sense codons have been reassigned to semi-conserved amino acids in *E. coli* through selective pressure incorporation (SPI) methodologies that activate amino acid misincorporation in quiescent cells to minimize the toxic effects of codon ambiguity [20,21]. Moreover, *Euplotes crassus* tolerates the incorporation of two amino acids (selenocysteine and cysteine) at the UGA codon and the dual use of this codon can occur within the same gene [22]. These examples highlight high genetic code flexibility, but how natural variation in codon-amino acid assignments emerges and is selected as well as the consequences of engineering the genetic code remain unclear.

**Table 1:** Genetic code alterations in mitochondrial and nuclear genomes. These changes are phylogenetically independent and some of them occur more than once (adapted from [3]).

	Unassigned ➔ X	Sense ➔ Unassigned	Stop ➔ Sense	Sense ➔ Stop	Sense ➔ Sense
**Mitochondrial**					^Ser^AGG ➔ Lys
	AGA ➔ Gly		UGA ➔ Trp		^Ile^AUA ➔Met
	AGA ➔ Ser	^Arg^CGN ➔ UN	UAA ➔ Tyr	^Ser^UCA ➔ Stop	^Leu^CUN ➔ Thr
		^Ser^AGR ➔ UN	UAG ➔ Leu		^Arg^AGA ➔ Ser
	AGR ➔ Stop		UAG ➔ Ala		^Arg^AGG ➔ Ser
					^Lys^AAA ➔ Asn
					^Arg^AGA ➔ Gly
					^Arg^AGG ➔ Gly
**Nuclear**		^Arg^AGA ➔ UN	UGA ➔ Trp		
		^Ile^AUA ➔ UN	UGA ➔ Cys		^Leu^CUG ➔ Ser
		^Arg^CGG ➔ UN	UAR ➔ Gln		

## 2. Structural and Molecular Features of Non-Standard Genetic Codes

### 2.1. Nuclear Genetic Code Variation

Most codon reassignments have been linked to alterations in components of the translational machinery, namely tRNAs, aminoacyl-tRNA synthetases and the release factors that recognize stop codons [23].

In bacteria, reassignments appear to be restricted to the UGA stop codon and are associated with disappearance of RF2, which recognizes the UGA and UAA termination codons and mutant tRNAs that misread these codons. UGA has been reassigned to Trp in *Mycoplasma* spp. [7] and *Spiroplasma citri* [24]. Recent metagenomics studies and single-cell sequencing approaches revealed that the uncultivated bacteria *Candidatus* Hodgkinia cicadicola [25] and BD1-5 [26] also decode UGA as Trp, while SR1 bacteria [27] and Gracilibacteria [28] decode it as Gly.

Mollicutes with altered codes have two Trp-tRNA species, one with the canonical CCA anticodon to decode the UGG-Trp codon and the other with the UCA anticodon for decoding the UGA stop codon [29]. Since only UAA and UAG codons are used as termination codons, these species maintained the RF1 (responsible for the recognition of UAA and UAG) and eliminated RF2 [30]. Their small and AT-rich genomes (e.g., *Mycoplasma capricolum* AT content is ~75%) is likely to introduce important codon usage biases that may force the replacement of UGA for UAA codons. This renders RF2 dispensable as RF1 alone is able to recognize the remaining UAA and UAG termination codons [31].

Conversely, the reassignment of UGA to Trp exists in the GC-rich (~60%) genome of *Candidatus* Hodgkinia cicadicola, where the RF2 is absent. In this case, there is only one Trp-tRNA with an UCA anticodon, suggesting that its gene arose from mutation and not from tRNA gene duplication. Authors proposed that this identity change arose from codon ambiguity initiated by the emergence of a mutant Trp-tRNA that could decode the UGA codon. This tRNA competed with RF2 for the UGA stop codon, which eventually led to the dispensability of RF2 and to further tRNA mutations to refine its new decoding properties. The Hodgkinia genome adapted to the new codon usage by replacing the old UGA stop codons with UAA and UAG codons and by substituting some of the UGG codons for UGA [25].

The reassignment of UGA to Gly in SR-1 bacteria present in the human microbiome is also accompanied by the loss of RF2. Apart from the canonical Gly-tRNA_UCC_ which decodes GGN-Gly codons, its genome also encodes an additional Gly-tRNA_UCA_. Although the D and anticodon arms of this unusual tRNA are divergent from the canonical Gly-tRNA, it maintains the major identity elements for glycylation by the GlyRS [27].

Little is known about codon alterations in phages, but several reports suggest reassignment of the UGA stop codon to Trp [32] and to Gly, and the UAG stop to Ser and Gln [33]. Since bacteria appear to reassign only UGA codons, the use of a divergent code in bacteriophages has important implications. It has been suggested that differences between viral and host genetic codes constitute a barrier to infection, because phages are deeply dependent on the translation machinery of their hosts [32,34], but these phases encode Gln-tRNA_CUA_ or Ser-tRNA_CUA_ and RF2 to translate UAG codons. Since bacteria that use UGA codons as sense codons erased RF2, such phages are able to infect their hosts [33].

In eukaryotes, termination codons are also reassigned by the cytoplasmic translational machinery. These alterations are again associated with misreading tRNAs, aminoacyl-tRNA synthetases and release factors [23]. Since eukaryotes have only one release factor (eRF1) to decode the UAA, UAG and UGA stop codons and this factor has three well defined domains [35]: domain 1 is responsible for stop codon recognition [36], domain 2 is associated with peptide hydrolysis [37] and domain 3 interacts with eRF3, a GTPase which stimulates termination activity [38], changes in the stop codon recognition domain, *i.e.* domain-1, are associated with stop codon reassignment.

**Figure 1:**
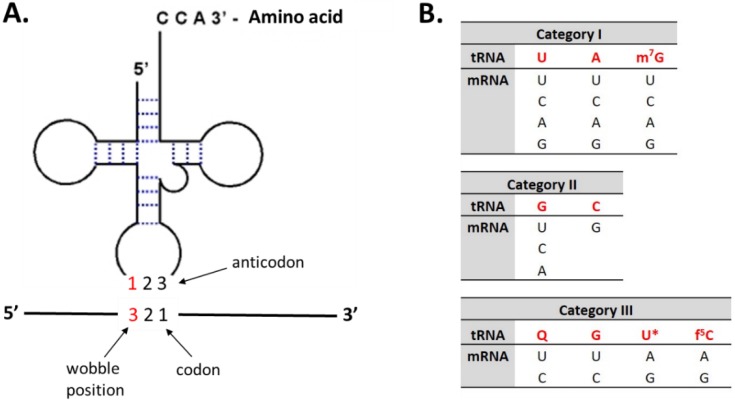
(**A**) An expanded wobble rule; (**B**) Possible pairings between the wobble nucleoside of tRNA and the codon third nucleoside of mRNA found in animal mitochondria. U *: cmnm^5^(s^2^)U, mnm^5^U, τm^5^U, τm^5^s^2^U (adapted from [64]).

UAR stop codons have been reassigned to Gln in the diplomonad *Hexamita inflate* [39], the oxymonad *Streblomastix strix* [40] and in several dasycladalean, cladophoralean and trentepohlialean green algae [41]. Several species of ciliates use different deviant codes that arose independently. UAR stop codons have been reassigned to Gln in *Paramecium* [42], *Tetrahymena* [43], *Oxytricha*, *Loxodes* [44] and *Stylonychia* [45], and also to Glu in *Vorticella* and *Opisthonecta* [46]. The UGA stop codon has been reassigned to Cys in *Euplotes* spp. [47] and to Trp in *Blepharisma americanum* and *Colpoda* [48]. Decoding of UAR codons as Gln in *Tetrahymena thermophila* requires two additional Gln-tRNAs with the anticodons UUA and CUA while translation of the canonical CAR-Gln codons is accomplished by the usual Gln-tRNA_UUG_ [6]. On the other hand, *Euplotes* has only one gene for Cys-tRNA with a GCA anticodon and so the decoding of UGA codons requires an unusual G-A base pairing in the wobble position (Figure 1A) [49]. Apart from the emergence of suppressor tRNAs able to decode UGA or UAR codons, ciliate eRF1s must have altered stop codon recognition specificities.

Several studies implicated a series of modifications in domain 1 of divergent eRF1, particularly in the highly conserved TASNIKS and YCF motifs, which are involved in stop codon recognition (Table 2). However, substitutions across ciliate species are not alike and show different modes of stop codon specificity [50,51]. Ciliates that use UAR as sense codons terminate translation only at UGA codons and their eRF1 is UGA specific. Introduction of the divergent YCF motif of *Stylonychia* (QFMYFCGGKF) in the human eRF1 is sufficient to alter its specificity to UGA-only [52]. However, in both *Paramecium* and *Loxodes*, the divergent YCF alone is not sufficient and must act together with the altered TASNIKS motif to ensure UGA-only specificity [50,52]. Data is not consistent for *Tetrahymena* eRF1 *in vitro* and in *in vivo* studies. Chimeras of domains 2 and 3 of yeast eRF1 fused with the entire domain 1 of *Tetrahymena* result in UGA-only specificity *in vitro* [53], but it retains the ability to recognize all three codons *in vivo* [54]. Introduction of *Tetrahymena* TASNIKS and YCF motifs in human eRF1 does not alter recognition of UAA and UGA codons, but dramatically increases readthrough at UAG codons [50]. It has been suggested that *Tetrahymena* represents an ambiguous intermediate stage of the codon reassignment process as eRF1 retains the ability to recognize all three stop codons and reassignment is accomplished by competition from its suppressor Gln-tRNAs [54] that efficiently decode UAR codons as Gln [6]. Conversely, *Blepharisma* and *Euplotes* reassigned UGA stop codons to Cys and only UAR codons are recognized by their eRF1 as termination codons [55]. Both have a single substitution from Leu-126 to Ile in the YCF motif—YICDNKF. Introduction of this mutation in *S. cerevisiae* eRF1 dramatically increased the readthrough at UGA sites [50]. Another consistent substitution found in both genera is Ser-70 to Ala, which has been shown to increase UGA readthrough *in vivo*, while maintaining efficient termination at UAR codons. For the efficient discrimination of guanine in the second codon position, Ser-70 must be able to form a hydrogen bond with Ser-33 (GTS loop), whose interaction is lost upon substitution with alanine [56].

**Figure 2:**
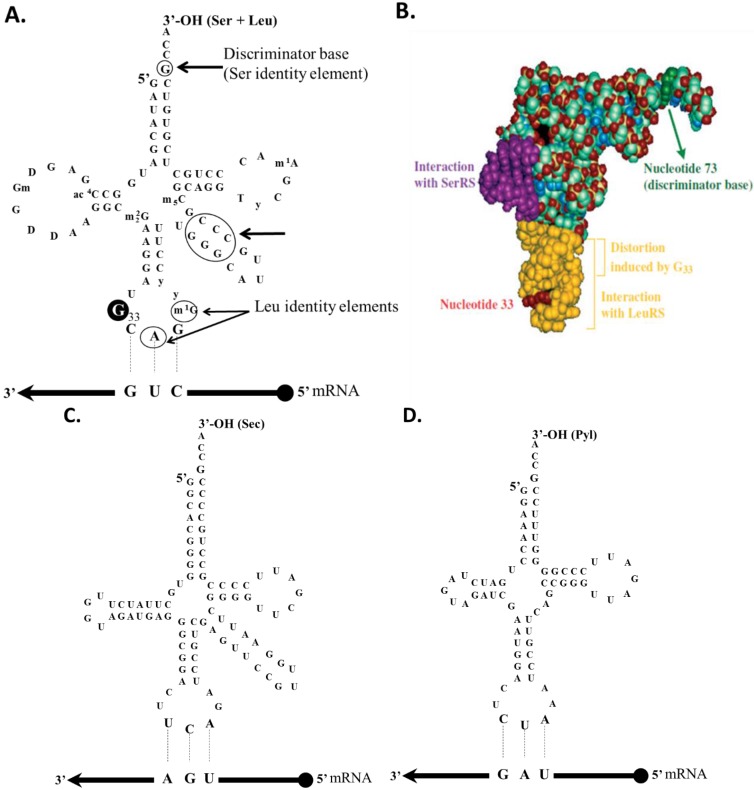
tRNA secondary structures. (**A**,**B**) A purine at position 33 (G33) in the *C. albicans* tRNA ^Ser^_CAG_ anticodon loop replaces a conserved pyrimidine found in all other tRNAs and is a key structural element in the reassignment of the CUG codon from leucine to serine. Two other nucleotides in the anticodon loop, A35 and G37, are important for leucylation, and the discriminator base, G73, functions as a negative identity determinant for leucyl-tRNA synthetase (A73 is required for leucylation); (**C**) tRNAs^Sec^ from all domains of life are unusual in both length (>90 nt) and structure. Most tRNAs have 7 bp in the acceptor stem and 5 in the TΨC arm, while eukaryal and archaeal tRNAs^Sec^ exhibit a 9 bp in the acceptor stem and 4 in the TΨC arm. Eukaryotic and archaeal tRNA^Sec^ species have 6 or 7 bp D-stems, respectively. Molecular modeling suggested that a 7 bp D-stem in archaeal tRNA^Sec^ would compensate for the short 4 bp T-stem thus allowing for the normal interaction between the D- and T-loops; (**D**) tRNAs^Pyl^ has a smaller D-loop (4–5 bp). Only one base is found between the acceptor and D-stems, rather than two bases, and the almost universally conserved G-purine sequence in the D-loop and TΨC sequence in the T loop are lacking. The anticodon stem forms with six, rather than five, base pairs, leaving only a very short (three base only) variable loop (adapted from [3]).

The only known sense-to-sense reassignment in nuclear genomes is found in several *Candida* species [5] where the CUG codon is reassigned from Leu to Ser, although its decoding *in vivo* still involves some degree of ambiguity [57,58,59]. This code alteration is mediated by a Ser-tRNA_CAG_ (Figure 2A,B) that is recognized by both SerRS and LeuRS [60,61]. It has the leucylation identity elements A_35_ and m^1^G_37_ and a U-to-G_33_ mutation which distorts the anticodon U-turn and lowers its leucylation and decoding efficiencies. The discriminator base is G_73_ which is a major identity element for serylation along with 3 GC pairs in the variable arm [60,61].

**Table 2:** Mutations in the highly conserved TASNIKS and YCF motifs of domain 1 of ciliate eRF1s, which alter stop codon recognition specificity and constitute an important step in codon reassignment (adapted from [51]).

	TASNIKS motif	YCF motif	S70
**Canonical codes**	TASNIKS	YLCDNKF	Ser
***Paramecium tetraurelia***	EAASIKD	YFCDPQF	Ser
***Loxodes striatus***	RAQNIKS	FLCENTF	Ala
***Oxytricha trifallax***	AAQNIKS	YFCGGKF	Ser
***Tetrahymena thermophila***	KATNIKD	YFCDSKF	Ser
***Stylonychia lemnae***	AAQNIKS	YFCGGKF	Ser
***Stylonychia mytilus***	AAQNIKS	YFCGGKF	Ser
***Euplotes octocarinatus/a***	TAESIKS	YICDNKF	Ala
***Euplotes octocarinatus/b***	TAVNIKS	YICDNKF	Ala
***Euplotes aediculatus/a***	TAESIKS	YICDNKF	Ala
***Euplotes aediculatus/b***	TAVNIKS	YICDNKF	Ala
***Blepharisma americanum***	KSSNIKS	YICDNKF	Ala
***Blepharisma japonica***	KSSNIKS	YICDNKF	Ala
***Blepharisma musculus***	KSSNIKS	YICDNKF	Ala

### 2.2. Mitochondrial Variations

Mitochondria show a significant diversity of codon identity reassignments, comprising nonsense-to-sense, sense-to-sense, sense-to-nonsense and sense-to-unassigned codon changes [62]. Alterations appear to be facilitated due to their reduced genome size and complexity, which encodes only a small set of essential genes. Also, their genomes tend to be strongly biased as they are AT-rich [62]. They encode only a small set of tRNAs (for example, human mtDNAs encode 22 tRNA species [63] and thus each tRNA can read two to four codons in a four codon-box by expanded wobbling (Figure 1). For example, the presence of an unmodified U at anticodon position 34 (wobble) enables pairing with N-ending codons, allowing for decoding four codons in codon-boxes. Also, several modified nucleosides in the first and second position of the anticodon play critical roles in mitochondrial decoding [64].

Termination codons have been reassigned to different amino acids in mitochondria. The UAA codon is decoded as Tyr in the mitochondria of the nematode *R. similis* [65]. UAG codons are decoded as tyrosine by an unusual Tyr-tRNA_CUA_ in calcareous sponges [66], but in green algae its meaning has changed to Ala or Leu [67]. The most frequent reassignment involves decoding of the UGA stop as Trp [68]. This change is mediated by a Trp-tRNA with the anticodon UCA, where its wobble position carries a modified uridine. Modifications can be 5-carboxymethylaminomethyluridine (cmnm^5^U), 5-carboxymethylaminomethyl(2-thio)uridine (cmnm^5^s^2^U) or 5-taurinomethyluridine (τm^5^U) and they expand the decoding capacity to R-ending codons, enabling the decoding of UGG and UGA codons as Trp [69].

Sense codons also change identity in mitochondria and some are unassigned as they are not present in the mtDNA. Insertion of Met at Ile AUA codon is frequent in most metazoans. In mammalians, this identity change is mediated by a Met-tRNA_CAU_ with a modified C in the wobble position to 5-formylcytidine (f^5^C) [70], which enables decoding of both AUG and AUA codons [71]. Ascidian Met-tRNA has a τm^5^U modification in the same position [69]. The AAA-Lys codon is translated as asparagine in echinoderms and platyhelminths [72]. In starfish mitochondria, a single Asn-tRNA_GΨU_ with a modification to pseudouridine (Ψ) in the second position of the anticodon decodes the canonical AAY-Asn codons and the AAA-Lys codon. Also, its Lys-tRNA has a CUU anticodon, instead of GUU, which restricts its decoding to AAG only [73].

Mitochondria of the yeast species *Saccharomyces*, *Nakaseomyces* and *Vanderwaltozyma* decode the four Leu-CUN codons as threonine [74]. This alteration is associated with the loss of the Leu-tRNA_UAG_ capable of decoding the CUN codons and the appearance of a mutant Thr-tRNA_UAG_ with an unmodified U at the wobble position which enables recognition of all four nucleotides at the third codon position [64]. Interestingly, this Thr-tRNA has evolved from a His-tRNA_GUG_ due to loss of its typical guanosine at position -1 and substitution of the discriminator base C73 to A73 (critical identity elements for the HisRS) [75], and by addition of an adenosine at position 35. Consequently, its anticodon loop has 8-nt and is a substrate for the yeast ThrRS [76]. On the other hand, the yeast *Ashbya gossypii* decodes the CUU and CUA codons as Ala using an Ala-tRNA_UAG_ [77]. It was proposed that this tRNA evolved from the later Thr-tRNA_UAG_ through reduction of the anticodon loop (major identity element to *S. cerevisiae* ThrRS [78]) and introduction of a G3:U70 base pair which is a major identity element for the AlaRS [75].

Arginine AGA and AGG codons change identity very often and have different meanings, namely Ser [79], Gly [80] or stop [63]. Mitochondria that reassigned AGR codons lack the Arg-tRNA_UCU_ gene, which has been proposed as the initial step for these reassignments [68]. In the absence of the competitor Arg-tRNA_UCU_, the AGA codon is captured by a Ser-tRNA_GCU_ [81]. In *Drosophila*, AGG codons are absent and only AGA codons are decoded by the Ser-tRNA_GCU_ which has an unmodified G at the wobble position [82]. In squid and starfish mitochondria, the wobble position of Ser-tRNA_GCU_ is methylated to m^7^G_34_ which expands its capacity to read AGR-Arg codons, inserting serine at these sites [83]. On the other hand, the wobble position of Ser-tRNA of *Ascaris* mitochondria is occupied by an unmodified U [84], which allows decoding of AGN codons as Ser [81]. In ascidian mitochondria, AGR codons are decoded as Gly by a Gly-tRNA_UCU_ with a modification in the wobble position to τm^5^U [69]. Although the majority of changes are associated to the codon pair simultaneously, some arthropods and also the nematode *R. compacta* decode the AGG codon as Lys and AGA as Ser. These species have an unmodified Ser-tRNA_GCU_ for AGA codons and a Lys-tRNA with a CUU anticodon instead of the typical UUU anticodon, which is thought to recognize the AGG codons at low efficiency [85]. Interestingly, the appearance of this atypical Lys-tRNA_CUU_ restricts recognition of AAA-Lys codons, which has been correlated with its reassignment to Asn by Asn-tRNA_GUU_, in this case and in other species that do not use the AGG codon as Lys (e.g., in echinoderms) [73].

Another codon that is reassigned to stop is the UCA-Ser codon of the green alga *Scenedesmus obliquus* [86]. Both have in common the absence of the cognate tRNA that would recognize AGR or UCA codons, namely Arg-tRNA_UCU_ [68] and Ser-tRNA_UGA_, respectively. Since Ser-tRNA_UGA_ is responsible for decoding the UCN-Leu codon-box, *S. obliquus* has a Ser-tRNA_GGA_ to decode the other UCU and UCC codons, and UCG is an unassigned codon [86]. Termination codons have also been reassigned in mitochondria. The reassignment of the UGA codon to Trp happens in all animal mitochondria [64]. These reassignments require changes in the release factors, but the termination mechanism in mitochondria remains an unsolved question. Four different homologues to bacterial release factors have been found in human mitochondrial systems: mtRF1, mtRF1a, ICT1 and C12orf65 [87]. To date, none of these factors have shown specific UGA release activity. Although molecular dynamics simulations have proposed that mtRF1 may behave like RF1 [88] or that it may rescue stalled ribosomes with empty A-sites [89], its function remains elusive since no *in vitro* release activity has been found for any termination codon, including AGR codons [90]. mtRF1a has *in vitro* and *in vivo* release activity in response to UAG and UAA stop codons, similarly to bacterial RF1 [91]. ICT1 is an integral member of the mitoribosome with codon-independent peptidyl-tRNA hydrolase activity [87], and is supposed to function as a multipurpose rescue factor for stalled ribosomes [90]. Regarding the use of AGR codons as termination codons in vertebrate mitochondria, one must consider the absence of the Arg-tRNA_UCU_ that decodes AGR codons [68]. Since it is expected that the ribosome stalls at these sites, ICT1 recognizes it and terminates translation at AGR sites [90].

### 2.3. Natural Expansion of the Genetic Code to 22 Amino Acids

Termination codons are also the target for the incorporation of the non-canonical amino acids selenocysteine (Sec), in a wide range of prokaryotes and eukaryotes [92], and pyrrolysine (Pyl) in archaeal Methanosarcina species [93], producing novel classes of proteins.

Incorporation of Sec in response to an in-frame UGA codon is achieved by complex recoding machinery that informs the ribosome not to stop at this position. The mechanism is distinct in prokaryotic and eukaryotic organisms, but there are some similarities. Both have a special Sec tRNA, which is a minor isoacceptor derived from a serine tRNA (Figure 2C). The other key players are SelB and SECIS (selenocysteine insertion sequence). Since Sec has its own tRNA^Sec^, biosynthesis begins with SerRS acylating tRNA^Sec^ with serine, producing Ser-tRNA^Sec^. Then, different enzymes convert Ser-tRNA^Sec^ into Sec-tRNA^Sec^: selenocysteine synthase (SelA) and selenophosphate synthetase (SelD) in bacteria and *O*-phosphoseryl-tRNA kinase (PSTK) and Sep-tRNA:Sec-tRNA synthase (SepSecS) in archaea and eukarya [10,94]. Once the Sec-tRNA^Sec^ is available, recoding of UGA as Sec requires the presence of the translation elongation factor SelB. This factor binds to Sec-tRNA^Sec^ and forms the SelB.GTP.Sec-tRNA^Sec^ complex that is delivered to the ribosome. Studies performed by Bock and co-workers revealed that SelB must be complexed with the SECIS element for the correct interaction with the ribosome to occur [92]. Binding of the ternary complex to the SECIS structure induces a conformational change in SelB that enables codon–anticodon interaction between the Sec-tRNA^Sec^ and the UGA codon at the ribosomal A-site. Therefore, the SECIS element has a critical double function. It converts SelB into a “competent state” that gives SelB a strong competitive advantage relative to the release factor for decoding UGA. Simultaneously, it prevents normal UGA termination codons from being decoded as Sec by the SELB.GTP.Sec-tRNA^Sec^ ternary complex. The dual properties of SelB and SECIS ensure that only UGA codons in selenoprotein mRNAs are recoded [9].

While Sec is generated by a pretranslational modification of Ser-tRNA^Sec^ (Figure 2D), pyrrolysine (Pyl) is directly attached to tRNA^Pyl^_CUA_ by PylRS in response to an in-frame UAG codon in the *Methanosarcina barkeri* monomethylamine methyltransferase gene [12]. These are methane-producing organisms and Pyl is necessary for methane biosynthesis from methylamines. Indeed, the three different methyltransferases that initiate methanogenesis from different methylamines have genes with an in-frame UAG codon which is translated as pyrrolysine [11,93]. The mechanism for Pyl insertion requires a tRNA^Pyl^ (tRNA^Pyl^_CUA_) and a pyrrolysyl-tRNA synthetase (PylRS). The PylRS is considered the 21st AARS, since it charges specifically Pyl to tRNA^Pyl^_CUA_ (lysine itself and its cognate tRNA^Lys^ are not substrates of this enzyme) [95]. Therefore, PylRS is the first example of a synthetase that is specific for a modified amino acid; PylRS and tRNA^Pyl^ form a naturally occurring AARS-tRNA pair that is effectively orthogonal to the canonical genetic code [11].

Several mechanisms for Sec and Pyl insertion in protein sequences are present in different organisms, but context dependency is the universal feature of these occurrences and they can be regarded as preprogrammed modifications of canonical decoding rules.

## 3. Genetic Code Expansion for Co-Translational Protein Engineering

The study of structural and molecular features of non-standard genetic codes, in addition to support models for codon reassignment theories (reviewed in [96,97]), also provides useful information for synthetic rewriting of genetic codes.

Incorporation of non-canonical amino acids (ncAAs), in particular, the isostructural ncAAs which are recognized by the endogenous host cell machinery, has been possible by replacement of canonical amino acids (cAAs) using a supplementation-based incorporation method (SPI). This approach uses auxotrophic strains for one of the common 20 canonical amino acids (cAAs) to replace a specific cAA with a ncAA. The method exploits the natural tolerance of the host AARSs to the isostructural ncAAs, which allows the concurrent exchange of many residues in a target protein by sense-codon reassignment [98]. Although the overall replacement of a cAA by a ncAA cannot be tolerated during exponential growth, non-dividing cells are viable and are able to overexpress proteins that contain the ncAA. The diversity of amino acid analogs that can be incorporated using this approach has been increased through AARS overexpression, active-site engineering and editing domain mutations [99]. Numerous examples of applications of this technique are available, including the replacement of methionine with selenomethionine to introduce a heavy atom into proteins for crystallographic phasing experiments [100] and, in other cases, methionine or phenylalanine have been replaced by alkyne-containing ncAA analogs to track newly synthesized proteins [101].

**Figure 3:**
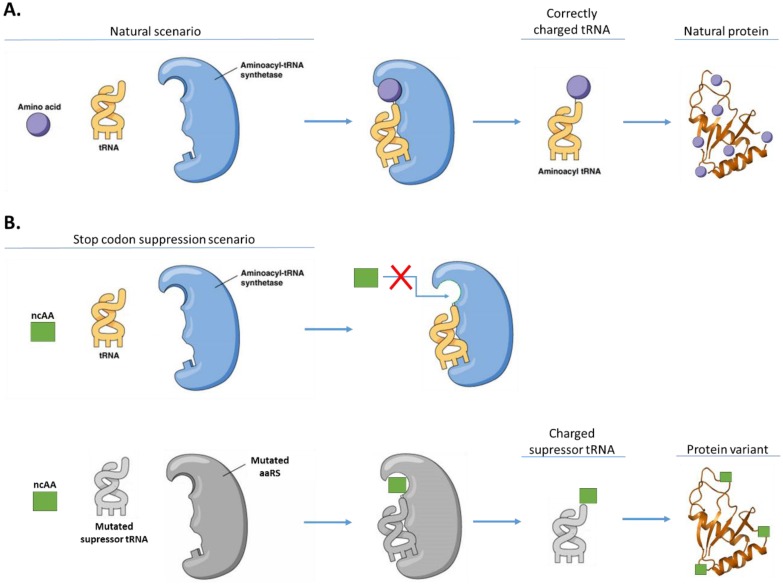
(**A**) Aminoacylation with canonical amino acids. tRNA aminoacylation is catalyzed by the corresponding aminoacyl-tRNA synthetase responsible for charging the tRNA with the cognate amino acid; (**B**) Stop codon suppression methods use heterologous orthogonal AARS:tRNA pairs to incorporate an orthogonal amino acid in response to a stop or quadruplet codon. This orthogonal amino acid is not a substrate for the endogenous tRNA and AARS (adapted from [104]).

As for orthogonal ncAAs (that do not participate in conventional translation), they have been added by site-specific incorporation in response to stop or quadruplet codons (stop codon suppression, SCS) using orthogonal aminoacyl-tRNA synthetase:tRNA pairs (Figure 3) [102]. Orthogonal tRNAs and AARSs are constructed by following a series of conditions that contribute to the lack of cross-reactivity between the pair and the endogenous host synthetases, amino acids and tRNAs. Firstly, the tRNA cannot be recognized by the endogenous AARSs of the host, but must function efficiently in translation. Another crucial requirement for the tRNA is that it must deliver the ncAA in response to a unique codon that does not encode any of the 20 cAA (for example, a stop codon). Secondly, the orthogonal AARS must aminoacylate only the orthogonal tRNA and none of the endogenous tRNAs. This synthetase must also aminoacylate the tRNA with only the desired unnatural amino acid and no endogenous amino acid. Similarly, the ncAA cannot be a substrate for the endogenous synthetases. Finally, the ncAA must be efficiently transported into the cytoplasm when added to the growth medium, or biosynthesized by the host [103]. A number of heterologous AARS/tRNA pairs have been developed to expand the genetic code of *E. coli*, yeast and mammalian cells. For example, the *E. coli* GluRS/human initiator tRNA, the *E. coli* TyrRS/*E. coli* tRNATyr, the *E. coli* LeuRS/*E. coli* tRNALeu, and the *M. mazei* PylRS/*M. mazei* tRNAPyl pairs are all orthogonal in *S. cerevisiae* [102], demonstrating the potential of this methodology for synthetic biology.

### 3.1. Reassignment of Stop Codons

Stop codon suppression is the most frequently used method to incorporate ncAA into proteins *in vivo*. This approach comprises the use of an orthogonal aminoacyl-tRNA synthetase/tRNA pair, specifically developed to introduce ncAAs at the stop codon, and deletion of the corresponding release factor to increase suppression efficiency. One of the first successful reassignments was performed by Mukai and colleagues that reassigned the UAG (amber) codon to the ncAA iodotyrosine (3-iodo-l-Tyr) [19]. They started by mutagenizing the UAG stop codon to UAA in seven essential genes of *E. coli*, which allowed the deletion of the RF1-encoding *prfA* gene (release factor 1 terminates gene translation at UAA and UAG). Next, cells were supplied with an amber suppressor archaebacterial TyrRS/tRNA_CUA_ pair that inserted 3-iodo-l-Tyr when it encountered UAG, as demonstrated by the full-length expression of a target protein containing six copies of the UAG codon [19,105,106].

Recently, several groups applied a genome wide editing approach where the replacement of the amber stop codon occurs not only in essential genes but in all instances [34, 107, 108]. For example, Lajoie *et al.* used both multiplex automated genome engineering (MAGE) [109] and conjugative assembly genome engineering (CAGE) [107] to replace all known UAG stop codons in *E. coli* MG1655 with synonymous UAA codons. This allowed the deletion of RF1 and, therefore, elimination of termination at UAG codons. The resulting organism allowed them to reintroduce amber codons, along with an orthogonal translation machinery (episomal pEVOL) to permit efficient and site specific incorporation of *p*-azidophenylalanine (pAzF) and 2-naphthalalanine (NapA) into green fluorescent protein (GFP). This recoded organism exhibited increased resistance to T7 bacteriophage, suggesting that new genetic codes could facilitate increased viral resistance [34].

Although this approach is widely used nowadays, it is mostly applied in prokaryotic organisms because deletion of RF1 is not viable in yeast or mammalian cells [110]. Another limitation of this method concerns the nonsense mediated mRNA decay (NMD) mechanism that degrades mRNAs with premature stop codons, which significantly decreases protein yield [111].

### 3.2. Reassignment of Sense Codons

Although recent methods for protein engineering rely on the manipulation of the translation apparatus of the host, the simplest method exploits the close structural similarity between ncAA and a natural amino acid. Due to this similarity, the appropriate aminoacyl-tRNA synthetase is not able to distinguish between cAA and ncAA and permits non-specific charging of the ncAA onto tRNA. Consequently, the activated ncAA-tRNA is used in the translation process and the ncAA is incorporated in response to the sense codon encoding the corresponding cAA. The efficiency of this method is improved when competition from the canonical amino acid for the reassigned sense codon is limited. Auxotrophic bacterial hosts starved for the natural amino acid and supplemented with the ncAA are often used. The success of this strategy was first demonstrated by Cohen and Cowie when they took advantage of the relaxed substrate binding pocket of MetRS to completely replace the natural amino acid methionine by its analog selenomethionine in an *E. coli* methionine auxotroph [112]. Since then, many other sense codons have been reassigned to incorporate ncAAs into proteins via global substitution [99].

Complementary techniques to this approach have also been used, particularly the over-expression of the aminoacyl-tRNA synthetase of interest and attenuation of its hydrolytic editing activity [113]. For example, overexpression of valyl-tRNA synthetase (ValRS) in a valine auxotroph led to incorporation of one of the stereoisomers of 4,4,4-trifluorovaline (2S,3R-Tfv) in response to valine codons, as indicated by mass spectrometry [114]. Also, Yang and Tirrell showed that mutation of the conserved threonine residue to tyrosine (T252Y) in the editing domain of *E. coli* LeuRS led to the disruption of the editing activity of the LeuRS, which allowed the incorporation of several unsaturated, non-canonical amino acids in response to leucine codons [115].

Another methodology takes advantage of codons that are decoded by wobbling. At the third position of such codons, Us and Cs can be read by G in the anticodon of the corresponding tRNA while As and Gs can be read by a U or pseudouridine. Kwon *et al.* introduced an orthologous PheRS/tRNA_AAA_ pair from yeast into an *E. coli* Phe auxotrophic host and put a target gene under a strong inducible promoter. This gene contained the UUC codon at all desired Phe sites, and a UUU wobble codon was inserted at specific sites for 2-naphthylalanine. The yeast PheRS was able to activate 2-naphthylalanine and charged it on the yeast Phe-tRNA_AAA_, allowing for the production of a recombinant protein with 2-naphthylalanine [116].

Rare codons provide another method to introduce ncAAs into proteins. For example, the rare AGG arginine codon in *E. coli* has been reassigned to ncAAs using the PylRS/tRNAPyl_CCU_ pair. Since codon usage and tRNA gene content coevolved to match each other, the endogenous Arg-tRNA_CCU_ content is low, which allowed the ncAA-activated orthogonal tRNA_CCU_ to outcompete the former for the AGG codon. Zeng *et al.* showed that when *N*-alloc-lysine was used as a PylRS substrate, almost quantitative occupancy of *N*-alloc-lysine at an AGG codon site was achieved in minimal medium [117]. Recently, Mukai and colleagues demonstrated the *in vivo* reassignment of the AGG sense codon from arginine to l-homoarginine. A variant of the archaeal pyrrolysyl-tRNA synthetase (PylRS) was engineered in order to recognize l-homoarginine. The expression of this variant with the AGG-reading tRNA^Pyl^_CCU_ permitted the efficient incorporation into proteins of the arginine analog. Subsequently, all AGG codons in essential genes were eliminated and the bacterial ability to translate AGG into arginine was restricted in a temperature-dependent manner [118].

### 3.3. Quadruplet Codons

Another opportunity to expand codons for ncAAs emerged from the discovery of naturally occurring frameshift suppressor tRNAs, namely UAG*N* suppressors (N being A, G, C, or T) derived from Su7-encoding glutamine, ACC*N* suppressors derived from sufJ-encoding threonine and CAAA suppressors derived from tRNA^Lys^ and tRNA^Gln^ [119]. In these cases, four bases specify an amino acid in response to a mutant tRNA with an extra nucleotide in its anticodon loop (eight nucleotides instead of the standard seven), which leads to a reading frame shift and synthesis of a full length protein. Following this rationale, an orthogonal four-base suppressor tRNA/synthetase pair was generated from *Pyrococcus horikoshii* tRNA^Lys^ sequences. The mutant suppressor pair permitted the incorporation of l-homoglutamine into proteins in *E. coli* in response to the quadruplet codon AGGA [119].

Frequently, quadruplets target a rare codon to avoid competition of the native tRNA for the first three bases, which decreases the yield of the target protein with the ncAA. Since the endogenous tRNA is readily accepted by the native ribosome, several groups developed “orthogonal” ribosomes [120,121] that only recognize altered ribosome-binding sites (RBS). The presence of these mutant RBSs assures that only mRNAs containing those sequences are translated by the orthogonal ribosomes with reduced premature termination (ribo-X). This methodology generated orthogonal ribosomes with increased amber suppression on the desired mRNA, while native ribosomes sustained the standard level of amber suppression. Ribo-X were then evolved to increase the efficiency of translation of quadruplet codons (ribo-Q). Recently, a protein containing an azide and an alkyne was produced efficiently using this approach, which allowed the establishment of an internal cross-link [122]. The expectation is that ribo-Q might enable more ambitious alterations to proteins in the near future.

## 4. Conclusions and Perspectives

Genetic code alterations may be much more frequent than previously expected, as indicated by the diverse range of alterations found to date (Table 1) [3,123]. Low codon usage, codon unassignment, genome GC pressure, genome minimization, small proteome size and tRNA disappearance are essential players for the evolution of the genetic code [96, 124,125,126]. The Codon Capture theory posits that under biased genome AT or GC pressure, certain codons vanish from the polypeptide coding sequences (ORFeome). These unassigned codons lead to loss of functionality of the corresponding tRNAs, which can be eliminated by natural selection [125]. These erased codons may be reintroduced by genetic drift. Since GC content fluctuates over time, the erased codons can re-emerge, but they may lack cognate tRNAs. Cells that are able to capture these codons and convert them to sense codons have a growth advantage and the codon reassignment can be achieved. The codon capture theory is supported by the disappearance of the CGG codon in *Mycoplasma capricolum* (25% genome G + C) and the AGA and AUA codons in *Micrococcus luteus* (75% genome G + C) [127]. On the other hand, there are several other examples of codon reassignments in organisms where strong GC biases do not exist, and even cases of codon reassignments that appear against such bias; for example, reassignment of the leucine CUU and CUA codons to threonine in the AT rich genome of yeast mitochondria [128]. These codon reassignments are better explained by the Ambiguous Intermediate theory [62,124]. This theory postulates that ambiguous codon decoding provides an initial step for gradual codon identity change, and wild-type or mutant misreading tRNAs are the critical elements of codon reassignment. The appearance of mutant tRNAs with altered/expanded decoding properties allows the recognition and translation of non-cognate codons that are incorporated into proteins in competition with cognate ones. Consequently, statistical proteins are produced and, if this ambiguous codon translation is advantageous for the organism, the alternative codon interpretation is selected by natural selection, leading to a new arrangement of the code [124]. This theory is strongly supported by CUG reassignment from leucine to serine in fungi [4,129].

The incidence of genetic code alterations in mitochondria suggests that proteome size imposes strong negative pressure on codon reassignment. This is in line with the Genome Minimization hypothesis that posits that replication speed imposes a strong negative pressure on the mitochondrial genome, leading to selection of small size genomes [126]. This is supported by a study in human mitochondria where only 13 of the 900 proteins of its proteome are encoded by its genome [130]. Since nuclear encoded proteins are synthesized in the cytoplasm using the standard genetic code and are transported into the mitochondria using a signal peptide translocation system, their synthesis escapes the disruption caused by mitochondrial codon reassignments.

The three theories are not exclusive, since the ambiguous intermediate stage can be preceded by a decrease in the content of GC rich codons, so that codon reassignment might be driven by a combination of evolutionary mechanisms [131]. Additionally, the unpredicted existence of AARSs specific for the noncanonical amino acids pyrrolysine and *O*-phosphoserine [11] raised the possibility that other amino acids with particular functions might exist in still-uncharacterized genomes.

Detailed characterization of natural reassignments was a key step for developing efficient strategies to expand the code for production of proteins with novel biochemical properties. Due to the central importance of engineering proteins for both basic research and biopharmaceutical drug development, there are several established methods to accomplish the incorporation of non-natural amino acids. These can offer selective advantages beyond the evolution of proteins with only the canonical amino acids. One area that benefits from expanded genetic codes is the field of synthetic biology. Synthetic biologists have successfully engineered a wide range of functions into artificial gene circuits, generating switches, oscillators, filters, sensors, and cell-cell communicators with potential applications in medicine, biotechnology, bioremediation, and bioenergy [132]. For example, selective pressure incorporation (SPI) methodologies are currently being used to incorporate non-natural amino acids with reactive functional groups that are critical in site-specific derivatization of proteins for therapeutic purposes. Cho and colleagues reported the recombinant expression of human growth hormone (hGH) containing a site-specifically incorporated *para*-acetylphenylalanine (pAcF), which served as a chemical handle for conjugation to poly(ethylene glycol) (PEG) [133]. The resulting homogeneously mono-PEGylated hGH showed favorable pharmacodynamics and is being developed clinically [133]. Also, SPI methodologies allowed the purification and identification of 195 newly synthesized proteins in human embryonic kidney (HEK293) cells by orthogonal labeling of non-natural amino acids that were incorporated proteome-wide, following the removal of the corresponding natural amino acid [134].

More recently, Romesberg and colleagues surpassed the dependency on the four natural nucleotides A, T, G, and C [135] by using unnatural base pairs (UBPs) that allowed the incorporation of 152 additional non-canonical amino acids. The future will likely include a host for new applications based on these new technologies.

## References

[1] Crick F.H. (1968). The origin of the genetic code. J. Mol. Biol..

[2] Ibba M., Soll D. (2004). Aminoacyl-tRNAs: Setting the limits of the genetic code. Genes Dev..

[3] Santos M., Santos M.A., Cannarozzi G.M., Schneider A. (2012). Structural and molecular features of non-standard genetic codes. Codon Evolution: Mechanisms and Models.

[4] Butler G., Rasmussen M.D., Lin M.F., Santos M.A.S., Sakthikumar S., Munro C.A., Rheinbay E., Grabherr M., Forche A., Reedy J.L. (2009). Evolution of pathogenicity and sexual reproduction in eight Candida genomes. Nature.

[5] Sugita T., Nakase T. (1999). Non-universal usage of the leucine CUG codon and the molecular phylogeny of the genus Candida. Syst. Appl. Microbiol..

[6] Hanyu N., Kuchino Y., Nishimura S., Beier H. (1986). Dramatic events in ciliate evolution: Alteration of UAA and UAG termination codons to glutamine codons due to anticodon mutations in two Tetrahymena tRNAs. EMBO J..

[7] Yamao F., Muto A., Kawauchi Y., Iwami M., Iwagami S., Azumi Y., Osawa S. (1985). UGA is read as tryptophan in Mycoplasma capricolum. Proc. Natl. Acad. Sci. USA.

[8] Van der Gulik P.T., Hoff W.D. (2011). Unassigned codons, nonsense suppression, and anticodon modifications in the evolution of the genetic code. J. Mol. Evol..

[9] Allmang C., Krol A. (2006). Selenoprotein synthesis: UGA does not end the story. Biochimie.

[10] Yuan J., O’Donoghue P., Ambrogelly A., Gundllapalli S., Sherrer R.L., Palioura S., Simonovic M., Soll D. (2010). Distinct genetic code expansion strategies for selenocysteine and pyrrolysine are reflected in different aminoacyl-tRNA formation systems. FEBS Lett..

[11] Gaston M.A., Zhang L., Green-Church K.B., Krzycki J.A. (2011). The complete biosynthesis of the genetically encoded amino acid pyrrolysine from lysine. Nature.

[12] Krzycki J.A. (2005). The direct genetic encoding of pyrrolysine. Curr. Opin. Microbiol..

[13] Chin J.W. (2014). Expanding and reprogramming the genetic code of cells and animals. Annu. Rev. Biochem..

[14] Li F., Zhang H., Sun Y., Pan Y., Zhou J., Wang J. (2013). Expanding the genetic code for photoclick chemistry in E. coli, mammalian cells, and A. thaliana. Angew. Chem. Int. Ed. Engl..

[15] Lin S., Zhang Z., Xu H., Li L., Chen S., Li J., Hao Z., Chen P.R. (2011). Site-specific incorporation of photo-cross-linker and bioorthogonal amino acids into enteric bacterial pathogens. J. Am. Chem. Soc..

[16] Wang F., Robbins S., Guo J., Shen W., Schultz P.G. (2010). Genetic incorporation of unnatural amino acids into proteins in Mycobacterium tuberculosis. PLoS ONE.

[17] Greiss S., Chin J.W. (2011). Expanding the genetic code of an animal. J. Am. Chem. Soc..

[18] Teramoto H., Kojima K. (2014). Production of Bombyx mori silk fibroin incorporated with unnatural amino acids. Biomacromolecules.

[19] Mukai T., Hayashi A., Iraha F., Sato A., Ohtake K., Yokoyama S., Sakamoto K. (2010). Codon reassignment in the Escherichia coli genetic code. Nucleic Acids Res..

[20] Bacher J.M., Ellington A.D. (2001). Selection and characterization of Escherichia coli variants capable of growth on an otherwise toxic tryptophan analogue. J. Bacteriol..

[21] Bacher J.M., de Crecy-Lagard V., Schimmel P.R. (2005). Inhibited cell growth and protein functional changes from an editing-defective tRNA synthetase. Proc. Natl. Acad. Sci. USA.

[22] Turanov A.A., Lobanov A.V., Fomenko D.E., Morrison H.G., Sogin M.L., Klobutcher L.A., Hatfield D.L., Gladyshev V.N. (2009). Genetic code supports targeted insertion of two amino acids by one codon. Science.

[23] Knight R.D., Freeland S.J., Landweber L.F. (2001). Rewiring the keyboard: Evolvability of the genetic code. Nat. Rev. Genet..

[24] Stamburski C., Renaudin J., Bove J.M. (1992). Mutagenesis of a tryptophan codon from TGG to TGA in the cat gene does not prevent its expression in the helical mollicute Spiroplasma citri. Gene.

[25] McCutcheon J.P., McDonald B.R., Moran N.A. (2009). Origin of an alternative genetic code in the extremely small and GC-rich genome of a bacterial symbiont. PLoS Genet..

[26] Wrighton K.C., Thomas B.C., Sharon I., Miller C.S., Castelle C.J., VerBerkmoes N.C., Wilkins M.J., Hettich R.L., Lipton M.S., Williams K.H. (2012). Fermentation, hydrogen, and sulfur metabolism in multiple uncultivated bacterial phyla. Science.

[27] Campbell J.H., O’Donoghue P., Campbell A.G., Schwientek P., Sczyrba A., Woyke T., Soll D., Podar M. (2013). UGA is an additional glycine codon in uncultured SR1 bacteria from the human microbiota. Proc. Natl. Acad. Sci. USA.

[28] Rinke C., Schwientek P., Sczyrba A., Ivanova N.N., Anderson I.J., Cheng J.F., Darling A., Malfatti S., Swan B.K., Gies E.A. (2013). Insights into the phylogeny and coding potential of microbial dark matter. Nature.

[29] Citti C., Marechal-Drouard L., Saillard C., Weil J.H., Bove J.M. (1992). Spiroplasma citri UGG and UGA tryptophan codons: Sequence of the two tryptophanyl-tRNAs and organization of the corresponding genes. J. Bacteriol..

[30] Inagaki Y., Bessho Y., Osawa S. (1993). Lack of peptide-release activity responding to codon UGA in Mycoplasma capricolum. Nucleic Acids Res..

[31] Ohama T., Inagaki Y., Bessho Y., Osawa S. (2008). Evolving genetic code. Proc. Jpn. Acad. Ser. B Phys. Biol. Sci..

[32] Shackelton L.A., Holmes E.C. (2008). The role of alternative genetic codes in viral evolution and emergence. J. Theor. Biol..

[33] Ivanova N.N., Schwientek P., Tripp H.J., Rinke C., Pati A., Huntemann M., Visel A., Woyke T., Kyrpides N.C., Rubin E.M. (2014). Stop codon reassignments in the wild. Science.

[34] Lajoie M.J., Rovner A.J., Goodman D.B., Aerni H.R., Haimovich A.D., Kuznetsov G., Mercer J.A., Wang H.H., Carr P.A., Mosberg J.A. (2013). Genomically recoded organisms expand biological functions. Science.

[35] Song H., Mugnier P., Das A.K., Webb H.M., Evans D.R., Tuite M.F., Hemmings B.A., Barford D. (2000). The crystal structure of human eukaryotic release factor eRF1--mechanism of stop codon recognition and peptidyl-tRNA hydrolysis. Cell.

[36] Bertram G., Bell H.A., Ritchie D.W., Fullerton G., Stansfield I. (2000). Terminating eukaryote translation: Domain 1 of release factor eRF1 functions in stop codon recognition. RNA.

[37] Seit-Nebi A., Frolova L., Justesen J., Kisselev L. (2001). Class-1 translation termination factors: Invariant GGQ minidomain is essential for release activity and ribosome binding but not for stop codon recognition. Nucleic Acids Res..

[38] Cheng Z., Saito K., Pisarev A.V., Wada M., Pisareva V.P., Pestova T.V., Gajda M., Round A., Kong C., Lim M. (2009). Structural insights into eRF3 and stop codon recognition by eRF1. Genes Dev..

[39] Keeling P.J., Doolittle W.F. (1996). A non-canonical genetic code in an early diverging eukaryotic lineage. EMBO J..

[40] Keeling P.J., Leander B.S. (2003). Characterisation of a non-canonical genetic code in the oxymonad *Streblomastix strix*. J. Mol. Biol..

[41] Cocquyt E., Gile G.H., Leliaert F., Verbruggen H., Keeling P.J., De Clerck O. (2010). Complex phylogenetic distribution of a non-canonical genetic code in green algae. BMC. Evol. Biol..

[42] Caron F., Meyer E. (1985). Does Paramecium primaurelia use a different genetic code in its macronucleus?. Nature.

[43] Horowitz S., Gorovsky M.A. (1985). An unusual genetic code in nuclear genes of Tetrahymena. Proc. Natl. Acad. Sci. USA.

[44] Tourancheau A.B., Tsao N., Klobutcher L.A., Pearlman R.E., Adoutte A. (1995). Genetic code deviations in the ciliates: Evidence for multiple and independent events. EMBO J..

[45] Helftenbein E. (1985). Nucleotide sequence of a macronuclear DNA molecule coding for alpha-tubulin from the ciliate Stylonychia lemnae. Special codon usage: TAA is not a translation termination codon. Nucleic Acids Res..

[46] Sanchez-Silva R., Villalobo E., Morin L., Torres A. (2003). A new noncanonical nuclear genetic code: Translation of UAA into glutamate. Curr. Biol..

[47] Meyer F., Schmidt H.J., Plumper E., Hasilik A., Mersmann G., Meyer H.E., Engstrom A., Heckmann K. (1991). UGA is translated as cysteine in pheromone 3 of *Euplotes octocarinatus*. Proc. Natl. Acad. Sci. USA.

[48] Lozupone C.A., Knight R.D., Landweber L.F. (2001). The molecular basis of nuclear genetic code change in ciliates. Curr. Biol..

[49] Grimm M., Brunen-Nieweler C., Junker V., Heckmann K., Beier H. (1998). The hypotrichous ciliate Euplotes octocarinatus has only one type of tRNACys with GCA anticodon encoded on a single macronuclear DNA molecule. Nucleic Acids Res..

[50] Conard S.E., Buckley J., Dang M., Bedwell G.J., Carter R.L., Khass M., Bedwell D.M. (2012). Identification of eRF1 residues that play critical and complementary roles in stop codon recognition. RNA.

[51] Inagaki Y., Doolittle W.F. (2001). Class I release factors in ciliates with variant genetic codes. Nucleic Acids Res..

[52] Lekomtsev S., Kolosov P., Bidou L., Frolova L., Rousset J.P., Kisselev L. (2007). Different modes of stop codon restriction by the Stylonychia and Paramecium eRF1 translation termination factors. Proc. Natl. Acad. Sci. USA.

[53] Ito K., Frolova L., Seit-Nebi A., Karamyshev A., Kisselev L., Nakamura Y. (2002). Omnipotent decoding potential resides in eukaryotic translation termination factor eRF1 of variant-code organisms and is modulated by the interactions of amino acid sequences within domain 1. Proc. Natl. Acad. Sci. USA.

[54] Salas-Marco J., Fan-Minogue H., Kallmeyer A.K., Klobutcher L.A., Farabaugh P.J., Bedwell D.M. (2006). Distinct paths to stop codon reassignment by the variant-code organisms Tetrahymena and Euplotes. Mol. Cell Biol..

[55] Kervestin S., Frolova L., Kisselev L., Jean-Jean O. (2001). Stop codon recognition in ciliates: Euplotes release factor does not respond to reassigned UGA codon. EMBO Rep..

[56] Blanchet S., Rowe M., von der H.T., Fabret C., Demais S., Howard M.J., Namy O. (2015). New insights into stop codon recognition by eRF1. Nucleic Acids Res..

[57] Bezerra A.R., Simoes J., Lee W., Rung J., Weil T., Gut I.G., Gut M., Bayes M., Rizzetto L., Cavalieri D. (2013). Reversion of a fungal genetic code alteration links proteome instability with genomic and phenotypic diversification. Proc. Natl. Acad. Sci. USA.

[58] Gomes A.C., Miranda I., Silva R.M., Moura G.R., Thomas B., Akoulitchev A., Santos M.A. (2007). A genetic code alteration generates a proteome of high diversity in the human pathogen *Candida albicans*. Genome Biol..

[59] Santos M.A., Tuite M.F. (1995). The CUG codon is decoded in vivo as serine and not leucine in Candida albicans. Nucleic Acids Res..

[60] Santos M.A., Keith G., Tuite M.F. (1993). Non-standard translational events in Candida albicans mediated by an unusual seryl-tRNA with a 5'-CAG-3' (leucine) anticodon. EMBO J..

[61] Santos M.A., Perreau V.M., Tuite M.F. (1996). Transfer RNA structural change is a key element in the reassignment of the CUG codon in Candida albicans. EMBO J..

[62] Knight R.D., Landweber L.F., Yarus M. (2001). How mitochondria redefine the code. J. Mol. Evol..

[63] Anderson S., Bankier A.T., Barrell B.G., de Bruijn M.H., Coulson A.R., Drouin J., Eperon I.C., Nierlich D.P., Roe B.A., Sanger F. (1981). Sequence and organization of the human mitochondrial genome. Nature.

[64] Watanabe K., Yokobori S. (2011). tRNA Modification and Genetic Code Variations in Animal Mitochondria. J. Nucleic Acids.

[65] Jacob J.E., Vanholme B., Van Leeuwen T., Gheysen G. (2009). A unique genetic code change in the mitochondrial genome of the parasitic nematode Radopholus similis. BMC Res. Notes.

[66] Lavrov D.V., Pett W., Voigt O., Worheide G., Forget L., Lang B.F., Kayal E. (2013). Mitochondrial DNA of Clathrina clathrus (Calcarea, Calcinea): Six linear chromosomes, fragmented rRNAs, tRNA editing, and a novel genetic code. Mol. Biol. Evol..

[67] Hayashi-Ishimaru Y., Ohama T., Kawatsu Y., Nakamura K., Osawa S. (1996). UAG is a sense codon in several chlorophycean mitochondria. Curr. Genet..

[68] Sengupta S., Yang X., Higgs P.G. (2007). The mechanisms of codon reassignments in mitochondrial genetic codes. J. Mol. Evol..

[69] Suzuki T., Miyauchi K., Suzuki T., Yokobori S., Shigi N., Kondow A., Takeuchi N., Yamagishi A., Watanabe K. (2011). Taurine-containing uridine modifications in tRNA anticodons are required to decipher non-universal genetic codes in ascidian mitochondria. J. Biol. Chem..

[70] Moriya J., Yokogawa T., Wakita K., Ueda T., Nishikawa K., Crain P.F., Hashizume T., Pomerantz S.C., McCloskey J.A., Kawai G. (1994). A novel modified nucleoside found at the first position of the anticodon of methionine tRNA from bovine liver mitochondria. Biochemistry.

[71] Takemoto C., Spremulli L.L., Benkowski L.A., Ueda T., Yokogawa T., Watanabe K. (2009). Unconventional decoding of the AUA codon as methionine by mitochondrial tRNAMet with the anticodon f5CAU as revealed with a mitochondrial in vitro translation system. Nucleic Acids Res..

[72] Telford M.J., Herniou E.A., Russell R.B., Littlewood D.T. (2000). Changes in mitochondrial genetic codes as phylogenetic characters: Two examples from the flatworms. Proc. Natl. Acad. Sci. USA.

[73] Tomita K., Ueda T., Watanabe K. (1999). The presence of pseudouridine in the anticodon alters the genetic code: A possible mechanism for assignment of the AAA lysine codon as asparagine in echinoderm mitochondria. Nucleic Acids Res..

[74] Miranda I., Silva R., Santos M.A. (2006). Evolution of the genetic code in yeasts. Yeast.

[75] Giege R., Sissler M., Florentz C. (1998). Universal rules and idiosyncratic features in tRNA identity. Nucleic Acids Res..

[76] Su D., Lieberman A., Lang B.F., Simonovic M., Soll D., Ling J. (2011). An unusual tRNAThr derived from tRNAHis reassigns in yeast mitochondria the CUN codons to threonine. Nucleic Acids Res..

[77] Ling J., Daoud R., Lajoie M.J., Church G.M., Soll D., Lang B.F. (2014). Natural reassignment of CUU and CUA sense codons to alanine in Ashbya mitochondria. Nucleic Acids Res..

[78] Ling J., Peterson K.M., Simonovic I., Cho C., Soll D., Simonovic M. (2012). Yeast mitochondrial threonyl-tRNA synthetase recognizes tRNA isoacceptors by distinct mechanisms and promotes CUN codon reassignment. Proc. Natl. Acad. Sci. USA.

[79] Haen K.M., Lang B.F., Pomponi S.A., Lavrov D.V. (2007). Glass sponges and bilaterian animals share derived mitochondrial genomic features: A common ancestry or parallel evolution?. Mol. Biol. Evol..

[80] Yokobori S., Ueda T., Watanabe K. (1993). Codons AGA and AGG are read as glycine in ascidian mitochondria. J. Mol. Evol..

[81] Yokobori S., Suzuki T., Watanabe K. (2001). Genetic code variations in mitochondria: tRNA as a major determinant of genetic code plasticity. J. Mol. Evol..

[82] Tomita K., Ueda T., Ishiwa S., Crain P.F., McCloskey J.A., Watanabe K. (1999). Codon reading patterns in Drosophila melanogaster mitochondria based on their tRNA sequences: A unique wobble rule in animal mitochondria. Nucleic Acids Res..

[83] Tomita K., Ueda T., Watanabe K. (1998). 7-Methylguanosine at the anticodon wobble position of squid mitochondrial tRNA(Ser)GCU: Molecular basis for assignment of AGA/AGG codons as serine in invertebrate mitochondria. Biochim. Biophys. Acta.

[84] Watanabe Y., Tsurui H., Ueda T., Furushima R., Takamiya S., Kita K., Nishikawa K., Watanabe K. (1994). Primary and higher order structures of nematode (Ascaris suum) mitochondrial tRNAs lacking either the T or D stem. J. Biol. Chem..

[85] Abascal F., Posada D., Knight R.D., Zardoya R. (2006). Parallel evolution of the genetic code in arthropod mitochondrial genomes. PLoS Biol..

[86] Kuck U., Jekosch K., Holzamer P. (2000). DNA sequence analysis of the complete mitochondrial genome of the green alga Scenedesmus obliquus: Evidence for UAG being a leucine and UCA being a non-sense codon. Gene.

[87] Richter R., Rorbach J., Pajak A., Smith P.M., Wessels H.J., Huynen M.A., Smeitink J.A., Lightowlers R.N., Chrzanowska-Lightowlers Z.M. (2010). A functional peptidyl-tRNA hydrolase, ICT1, has been recruited into the human mitochondrial ribosome. EMBO J..

[88] Lind C., Sund J., Aqvist J. (2013). Codon-reading specificities of mitochondrial release factors and translation termination at non-standard stop codons. Nat. Commun..

[89] Huynen M.A., Duarte I., Chrzanowska-Lightowlers Z.M., Nabuurs S.B. (2012). Nabuurs, Structure based hypothesis of a mitochondrial ribosome rescue mechanism. Biol. Direct.

[90] Akabane S., Ueda T., Nierhaus K.H., Takeuchi N. (2014). Ribosome rescue and translation termination at non-standard stop codons by ICT1 in mammalian mitochondria. PLoS Genet..

[91] Nozaki Y., Matsunaga N., Ishizawa T., Ueda T., Takeuchi N. (2008). HMRF1L is a human mitochondrial translation release factor involved in the decoding of the termination codons UAA and UAG. Genes Cells.

[92] Bock A., Forchhammer K., Heider J., Leinfelder W., Sawers G., Veprek B., Zinoni F. (1991). Selenocysteine: The 21st amino acid. Mol. Microbiol..

[93] Srinivasan G., James C.M., Krzycki J.A. (2002). Pyrrolysine encoded by UAG in Archaea: Charging of a UAG-decoding specialized tRNA. Science.

[94] Ambrogelly A., Palioura S., Soll D. (2007). Natural expansion of the genetic code. Nat. Chem. Biol..

[95] Blight S.K., Larue R.C., Mahapatra A., Longstaff D.G., Chang E., Zhao G., Kang P.T., Green-Church K.B., Chan M.K., Krzycki J.A. (2004). Direct charging of tRNA(CUA) with pyrrolysine *in vitro* and *in vivo*. Nature.

[96] Koonin E.V., Novozhilov A.S. (2009). Origin and evolution of the genetic code: The universal enigma. IUBMB Life.

[97] Moura G.R., Paredes J.A., Santos M.A. (2010). Development of the genetic code: Insights from a fungal codon reassignment. FEBS Lett..

[98] Link A.J., Mock M.L., Tirrell D.A. (2003). Non-canonical amino acids in protein engineering. Curr. Opin. Biotechnol..

[99] Voloshchuk N., Montclare J.K. (2010). Incorporation of unnatural amino acids for synthetic biology. Mol. Biosyst..

[100] Yang W., Hendrickson W.A., Crouch R.J., Satow Y. (1990). Structure of ribonuclease H phased at 2 A resolution by MAD analysis of the selenomethionyl protein. Science.

[101] Beatty K.E., Xie F., Wang Q., Tirrell D.A. (2005). Selective dye-labeling of newly synthesized proteins in bacterial cells. J. Am. Chem. Soc..

[102] Liu C.C., Schultz P.G. (2010). Adding new chemistries to the genetic code. Annu. Rev. Biochem..

[103] Wang L., Schultz P.G. (2001). A general approach for the generation of orthogonal tRNAs. Chem. Biol..

[104] Hoesl M.G., Budisa N. (2012). Recent advances in genetic code engineering in *Escherichia coli*. Curr. Opin. Biotechnol..

[105] Johnson D.B., Xu J., Shen Z., Takimoto J.K., Schultz M.D., Schmitz R.J., Xiang Z., Ecker J.R., Briggs S.P., Wang L. (2011). RF1 knockout allows ribosomal incorporation of unnatural amino acids at multiple sites. Nat. Chem. Biol..

[106] Mukai T., Hoshi H., Ohtake K., Takahashi M., Yamaguchi A., Hayashi A., Yokoyama S., Sakamoto K. (2015). Highly reproductive Escherichia coli cells with no specific assignment to the UAG codon. Sci Rep..

[107] Isaacs F.J., Carr P.A., Wang H.H., Lajoie M.J., Sterling B., Kraal L., Tolonen A.C., Gianoulis T.A., Goodman D.B., Reppas N.B. (2011). Precise manipulation of chromosomes *in vivo* enables genome-wide codon replacement. Science.

[108] Rovner A.J., Haimovich A.D., Katz S.R., Li Z., Grome M.W., Gassaway B.M., Amiram M., Patel J.R., Gallagher R.R., Rinehart J. (2015). Recoded organisms engineered to depend on synthetic amino acids. Nature.

[109] Wang H.H., Isaacs F.J., Carr P.A., Sun Z.Z., Xu G., Forest C.R., Church G.M. (2009). Programming cells by multiplex genome engineering and accelerated evolution. Nature.

[110] Nakamura Y., Ito K., Ehrenberg M. (2000). Mimicry grasps reality in translation termination. Cell.

[111] Wang Q., Wang L. (2008). New methods enabling efficient incorporation of unnatural amino acids in yeast. J. Am. Chem. Soc..

[112] Cohen G.N., Cowie D.B. (1957). Total replacement of methionine by selenomethionine in the proteins of *Escherichia coli*. C. R. Hebd. Seances Acad. Sci..

[113] Link A.J., Tirrell D.A. (2005). Reassignment of sense codons *in vivo*. Methods.

[114] Wang P., Fichera A., Kumar K., Tirrell D.A. (2004). Alternative translations of a single RNA message: An identity switch of (2S,3R)-4,4,4-trifluorovaline between valine and isoleucine codons. Angew. Chem. Int. Ed. Engl..

[115] Tang Y., Tirrell D.A. (2002). Attenuation of the editing activity of the Escherichia coli leucyl-tRNA synthetase allows incorporation of novel amino acids into proteins *in vivo*. Biochemistry.

[116] Kwon I., Kirshenbaum K., Tirrell D.A. (2003). Breaking the degeneracy of the genetic code. J. Am. Chem. Soc..

[117] Zeng Y., Wang W., Liu W.R. (2014). Towards reassigning the rare AGG codon in *Escherichia coli*. ChemBioChem.

[118] Mukai T., Yamaguchi A., Ohtake K., Takahashi M., Hayashi A., Iraha F., Kira S., Yanagisawa T., Yokoyama S., Hoshi H. (2015). Reassignment of a rare sense codon to a non-canonical amino acid in *Escherichia coli*. Nucleic Acids Res..

[119] Anderson J.C., Wu N., Santoro S.W., Lakshman V., King D.S., Schultz P.G. (2004). An expanded genetic code with a functional quadruplet codon. Proc. Natl. Acad. Sci. USA.

[120] Neumann H., Wang K., Davis L., Garcia-Alai M., Chin J.W. (2010). Encoding multiple unnatural amino acids via evolution of a quadruplet-decoding ribosome. Nature.

[121] Wang K., Neumann H., Peak-Chew S.Y., Chin J.W. (2007). Evolved orthogonal ribosomes enhance the efficiency of synthetic genetic code expansion. Nat. Biotechnol..

[122] Chen I.A., Schindlinger M. (2010). Quadruplet codons: One small step for a ribosome, one giant leap for proteins: an expanded genetic code could address fundamental questions about algorithmic information, biological function, and the origins of life. Bioessays.

[123] Santos M.A., Moura G., Massey S.E., Tuite M.F. (2004). Driving change: The evolution of alternative genetic codes. Trends Genet..

[124] Schultz D.W., Yarus M. (1996). On malleability in the genetic code. J. Mol. Evol..

[125] Osawa S., Jukes T.H. (1989). Codon reassignment (codon capture) in evolution. J. Mol. Evol..

[126] Andersson S.G., Kurland C.G. (1995). Genomic evolution drives the evolution of the translation system. Biochem. Cell Biol..

[127] Osawa S., Jukes T.H., Watanabe K., Muto A. (1992). Recent evidence for evolution of the genetic code. Microbiol. Rev..

[128] Osawa S., Collins D., Ohama T., Jukes T.H., Watanabe K. (1990). Evolution of the mitochondrial genetic code. III. Reassignment of CUN codons from leucine to threonine during evolution of yeast mitochondria. J. Mol. Evol..

[129] Ohama T., Suzuki T., Mori M., Osawa S., Ueda T., Watanabe K., Nakase T. (1993). Non-universal decoding of the leucine codon CUG in several Candida species. Nucleic Acids Res..

[130] Elstner M., Andreoli C., Ahting U., Tetko I., Klopstock T., Meitinger T., Prokisch H. (2008). MitoP2: An integrative tool for the analysis of the mitochondrial proteome. Mol. Biotechnol..

[131] Massey S.E., Moura G., Beltrao P., Almeida R., Garey J.R., Tuite M.F., Santos M.A. (2003). Comparative evolutionary genomics unveils the molecular mechanism of reassignment of the CTG codon in *Candida* spp.. Genome Res..

[132] Lu T.K., Khalil A.S., Collins J.J. (2009). Next-generation synthetic gene networks. Nat. Biotechnol..

[133] Cho H., Daniel T., Buechler Y.J., Litzinger D.C., Maio Z., Putnam A.M., Kraynov V.S., Sim B.C., Bussell S., Javahishvili T. (2011). Optimized clinical performance of growth hormone with an expanded genetic code. Proc. Natl. Acad. Sci. USA.

[134] Dieterich D.C., Link A.J., Graumann J., Tirrell D.A., Schuman E.M. (2006). Selective identification of newly synthesized proteins in mammalian cells using bioorthogonal noncanonical amino acid tagging (BONCAT). Proc. Natl. Acad. Sci. USA.

[135] Malyshev D.A., Dhami K., Quach H.T., Lavergne T., Ordoukhanian P., Torkamani A., Romesberg F.E. (2012). Efficient and sequence-independent replication of DNA containing a third base pair establishes a functional six-letter genetic alphabet. Proc. Natl. Acad. Sci. USA.

